# Perovskite solar cells: must lead be replaced – and can it be done?

**DOI:** 10.1080/14686996.2018.1460176

**Published:** 2018-05-24

**Authors:** Qi Zhang, Feng Hao, Jianbao Li, Yangying Zhou, Yaxuan Wei, Hong Lin

**Affiliations:** a State Key Laboratory of New Ceramics & Fine Processing, School of Materials Science and Engineering, Tsinghua University, Beijing, China; b School of Materials and Energy, University of Electronic Science and Technology of China, Chengdu, China; c State Key Laboratory of Marine Resource Utilization in South China Sea, Materials and Chemical Engineering Institute, Hainan University, Haikou, China

**Keywords:** Low-lead, metal-halide perovskite, toxicity, substitution, 40 Optical, magnetic and electronic device materials, 209 Solar cell / Photovoltaics

## Abstract

Perovskite solar cells have recently drawn significant attention for photovoltaic applications with a certified power conversion efficiency of more than 22%. Unfortunately, the toxicity of the dissolvable lead content in these materials presents a critical concern for future commercial development. This review outlines some criteria for the possible replacement of lead by less toxic elements, and highlights current research progress in the application of low-lead halide perovskites as optically active materials in solar cells. These criteria are discussed with the aim of developing a better understanding of the physio-chemical properties of perovskites and of realizing similar photovoltaic performance in perovskite materials either with or without lead. Some open questions and future development prospects are outlined for further advancing perovskite solar cells toward both low toxicity and high efficiency.

## Graphical abstract

**Figure:**
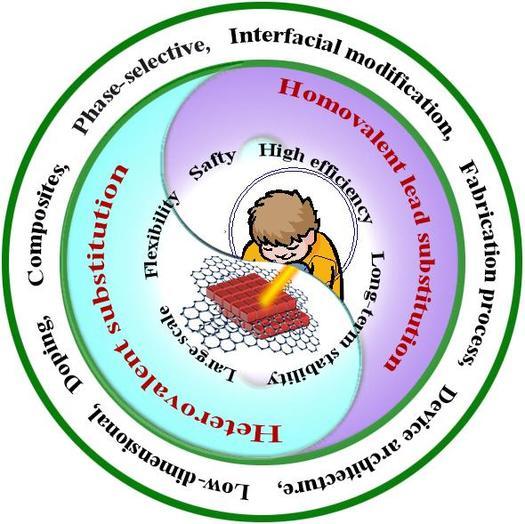

## 1. Introduction

Perovskite solar cells (PSCs), for use in superior photovoltaic (PV) devices with a high power conversion efficiency (PCE) and low cost in third-generation PV technologies, have undergone rapid progress during the last decade as a result of their numerous advantages including low density, and flexibility, as well as cost-effective production [1–5]. Such unique advantages have contributed directly to the competitiveness of PSCs versus earlier PV technologies based on commercial silicon (20%), GaAs (18.4%), cadmium telluride (CdTe, 19.6%), and copper indium gallium selenide/sulfide (CIGS, 19.6%) where the figures in parenthesis indicate the maximum PCE of commercial devices for each, and have helped to drive a sustained research interest toward alternative PV materials produced with cost-competitive, facile, and environmentally friendly technologies [6–8]. Through their role in the exploitation and development of novel materials, device architectures, and fabrication technologies, the development of PSCs has resulted in a “catfish effect” for other PV technologies [9,10]. It is noteworthy that the maximum PCE of PSCs has been improved from 3.8 to 22.7% in just 7 years. This great success essentially arises from the exceptional optoelectronic properties of semiconducting halide perovskites, namely a high optical absorption coefficient, a tunable band gap, long carrier recombination lifetimes, and a high electron/hole mobility and transmission quality, along with small electron/hole effective masses and exciton binding energy [11–19].

**Figure 1.**
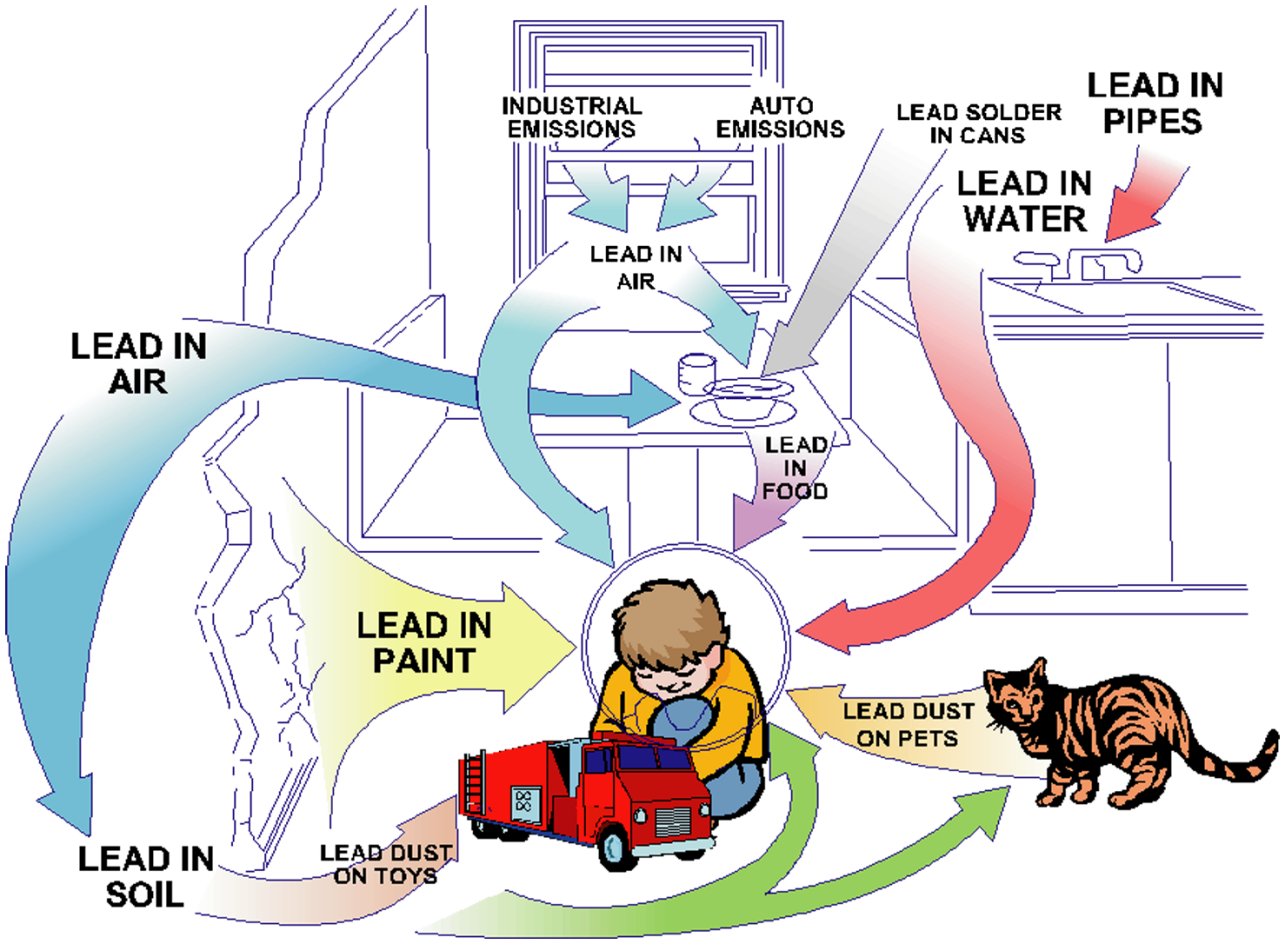
Environmental sources of childhood lead exposure [20].

Note: Soil, water, air, food, paint, toys, and pets are reported as the most important potential lead exposure sources for children.

Despite the impressive progress in lead-halide perovskite photovoltaics, the commercialization of PSCs still requires several challenges related to stability upon prolonged exposure to light, humidity, and high temperature to be addressed, as well as the development of suitable large-scale manufacturing processes. In addition, all current PSCs with high PCE contain the harmful element lead, raising a serious environmental concern for future commercial development. Although lead is present in common batteries, and equally toxic elements such as cadmium can also be found in commercial PV materials, occupational and environmental exposures to these toxic elements remains a serious problem in many developing and industrializing countries, and even in some developed countries. Once soil or water is polluted, lead and its compounds can result in long-term environmental damage and serious harm to human health on account of the high toxicity of lead, combined with a long degradation lifetime and stability in the ecosystem. In humans, lead is harmful to the nervous and reproductive systems and to the hematopoietic and renal organs, mainly as a result of increased oxidative stress. Moreover, it has been shown that the absorption of lead can be as high as 70% for children, while lead absorption is lower than 20% for adults, as a result of the numerous potential sources of exposure, according to U.S. Department of Health and Human Services, as shown in Figure 1. The presence of lead in the human body often remains undetected and can initially present no obvious symptoms, resulting in learning disabilities, behavioral problems, malformed bones, slow growth, seizures, coma, and even death. An important question therefore is whether it is possible to fully replace lead by less toxic elements in PSCs, without compromising on efficiency and stability, or whether is it the case that lead is absolutely essential for their performance. Some scholars have suggested that one viable option for reducing the lead hazard in PSCs is to develop effective encapsulation technologies against moisture and oxygen ingress, while another effective strategy could be to recycle these materials at the end of their lifetime. Despite such efforts, it is clear nevertheless that given the significant amount of lead that PVs presently contain this must ultimately be replaced, both for the sake of the environment and the long-term health of the human population. Nevertheless, can lead be replaced, and if so how? Alternatives to lead must fulfill some very stringent criteria to match the current performance of lead-halide perovskites, namely: (1) any alternatives should be low-cost, using abundant elements, be easily-recycled, and should form new compounds with a stable perovskite structure; (2) these new compounds should exhibit excellent optoelectronic properties; (3) such compounds should ideally additionally show superior performance as optically active materials (OAMs) in PSCs by combining material designs such as *in situ* substitution, semiconductor doping, non-stoichiometric perovskite structures, or low-dimensional or composite structures, with interfacial modification, along with device architecture optimization; (4) last but not least, it should be possible to manufacture these lead-free compounds from solution and moreover they should satisfy a variety of commercial requirements, such as flexibility, long-term stability, and scalability, in order to be competitive with current established PV technologies. Taking into consideration all these requirements, it is clear that elimination of lead from PSCs presents a formidable scientific challenge.

In this work we summarize the current research progress into low-lead halide perovskites. First we describe the respective merits both of lead-based halide perovskites and of potential alternatives via computational simulations, followed by a survey of the detailed materials design, interfacial modification, fabrication, and device architecture of low-lead PSCs based on tin, germanium, bismuth, antimony, indium, and transition metals (Cu, Mn). Finally, we outline some open questions and consider the future prospects for lead-free PSCs.

## 2. Perovskite crystal structure and lead replacement: theoretical calculations

For typical halide perovskites (ABX_3_), the A-sites are universally occupied by monovalent organic cations such as methylammonium (CH_3_NH_3_^+^ or MA^+^) and formamidinium (NH_2_CH=NH_2_^+^ or FA^+^), or inorganic alkali metal cation (Cs^+^), while the X positions, occupied by halogen anions or their mixtures, act to significantly influence the stability and optoelectronic properties of the perovskites. Empirical observations have suggested that the ideal tolerance factor, *t,* of ternary halide perovskites varies from 0.813 to 1.107, and this *t* value can readily determine whether a compound can form a cubic or cubic-like perovskite structure by adjustment of the halogen anions [21]. The B-sites, occupied by divalent metal ion (Pb^2+^, Ge^2+^, or Sn^2+^), provide numerous advantages according to the specific electronic structure, of high electrical mobility, a tunable band gap, mechanical and thermal stability, magnetic and dielectric transition, and mechanical plasticity, as well as structural and functional diversity. As lead halide perovskites exhibit a superior performance with a best certified PCE of ~22.7%, this success also provides a favorable reference for the fundamental understanding of the key properties, and for materials screening to exploit alternatives to lead. Such properties include (i) a strong and quickly-rising direct-gap optical transition between the valence Pb-s/X-p and conduction Pb-p states, (ii) a small exciton binding energy, allowing fast disengagement of optically generated electrons from holes, (iii) simultaneously low effective masses of electrons and holes, facilitating their transport, (iv) energetically shallow intrinsic defect levels, beneficial for bipolar conductivity and meanwhile minimizing carrier trapping and scattering, and (v) suitability of low-cost, non-vacuum solution routes for film deposition.

To determine whether the alternatives can replace lead or not, materials screening via computational simulations is of valuable help to avoid the expensive trial-and-error process of experimental exploration. These simulations have been guided by the specific optoelectronic properties and structure defects of PSCs. Generally speaking, as the electronic properties of perovskites are known to be strongly dependent on distortions of the crystal lattice, such as tilting and deformation of the octahedral, or ferroelectric distortions, the local density approximation (LDA) to density functional theory (DFT) [22,23] has been used to simulate band gaps, carrier mobilities, theoretical absorption spectra, electron/hole effective masses, and further calculate geometry optimizations, stability fields, and spin-orbit effects. Simulation tools for these studies include the Quantum Espresso Simulation Package (QESP) [24–26], the Vienna Ab Initio Simulation Package (VASP) [27–29], PBEsol [30], HSE06 [31], the Generalized Gradient Approximation (GGA) functional PBE [32], projector-augmented wave models [33], and Furthmüller [34] or spectroscopic limited maximum efficiency (SLME) methods [35]. In this section, discussion of less toxic alternatives to lead will be further classified into categories of homovalent or heterovalent lead substitution for a better understanding of the criteria for the future replacement of lead.

### 2.1. Homovalent lead substitution

Alternatives to lead should be metals admitting the oxidation state +2, and such elements constitute a significant part of the periodic table. As outer ns^2^ electrons of such ions have a critical impact on the modulation of optoelectronic properties, and as the Pb(*6s*)-orbital provides an inactive characteristic, the alternatives can originate from environment-friendly cations with similar outer shell *s*-orbitals, such as tin (II) (Sn^2+^) and germanium (II) (Ge^2+^). The PSCs developed using these alternatives have, to date, successfully demonstrated limited PCEs with best values of ~6.4% for MASnI_3_ [36] and ~0.2% for MAGeI_3_ [37]. In addition, empirical results have also indicated that exchanging the A-site cations for polar molecules with flexible and random orientation not only allows a large variety of lead-free perovskites to be produced, but also allows adjustment of the phase structure, optoelectronic properties and stability of these perovskites. A recent study predicted 14 Sn- and Ge-based perovskites with potential superior bulk-material-intrinsic PV performance using functionality-directed theoretical materials selection. However, only a few perovskites showed some PV performance, leaving a large scope for further exploration [38].

Additional theoretical and experimental studies on the partial replacement of Pb^2+^ by Sr^2+^ [39], Ca^2+^, and Cd^2+^, aimed at adjusting the electronic structure and carrier density of perovskites, provide a new strategy toward reducing the lead-related toxicity [40]. This strategy is not only suitable for homovalent lead substitution, but also applicable to heterovalent substitution, typically including In^3+^, Cu^+^, and Mn^+^. Taking into consideration the band gaps and stability as concurrent criteria, ions such as Mg^2+^, V^2+^, Mn^2+^, Ni^2+^, Cd^2+^, Hg^2+^, Ga^2+^, In^2+^ as well as Ge^2+^ and Sn^2+^ have been proposed as novel alternatives for the replacement of Pb^2+^ by Marina and co-workers. Among 25 possible compounds explored by these authors, a series of superior Mg-based halide perovskites such as CsMgI_3_, MAMgI_3_, and FAMgI_3_ with calculated band gaps of ~1.7, ~1.5, and ~0.9 eV, respectively, have been highlighted [41]. It can be noted, however, that this study does not cover all the potential low-toxicity elements as authors excluded rare earth elements and further neglected the consideration of polymorphs, oxidization, phase transition, and decomposition of these perovskites.

### 2.2. Heterovalent substitution

Heterovalent substitution is considered as another efficient strategy toward providing less toxic alternatives to lead. Specifically, the A_2_^I^B^I^B^III^X_6_ double perovskites show a controllable band gap, as well as ferroelectric, ferromagnetic, and multiferroic properties. All of these superior optoelectronic properties offer a benefit for PV applications [42]. The earliest research on double perovskites, using Cs_2_NaBiX_6,_ was reported in the 1970s [43–45]. As the field matures, researchers are increasingly replacing Na by Ag because of the electrical conductivity associated with the filled d^10^ shell and the free-electron-like behavior of the s^1^ shell, both of which improve the PV performance. The first reported A_2_^I^B^I^B^III^X_6_ double perovskites for PV application were developed in 2016, where the B^I^ and B^III^ sites can be occupied by monovalent noble metals Cu, Ag, and Au, and by trivalent metallic cations Bi and Sb, respectively [46]. This heterovalent substitution maintains total charge neutrality and allows for tunable band gaps of below 2.7 eV, spanning the visible and near-infrared optical spectrum. As an example of these double perovskites, Cs_2_BiAgCl_6_ with space group Fm$\overset{¯}{3}$m has an indirect band gap of ~2.2 eV, thus demonstrating convincingly this substitution criterion.

Further study on the specific electron configuration of the B^I^ groups can be naturally classified into three types: (I) the group IA elements (Na^+^, K^+^, and Rb^+^) that have an empty *s* shell and outmost *d* orbitals; (II) the group IB elements (Cu^+^, Ag^+^, and Au^+^) that have an empty *s* shell but full outmost *d* orbitals; and (III) the group IIIA elements (In^+^ and Tl^+^) that have full outmost *s* orbitals [47]. For most halide perovskites, as the X^VII^ group is changed from F^−^, Cl^−^, Br^−^ to I^−^, the band gap generally decreases. In contrast, as the B^III^ group is changed from Sb^3+^ to Bi^3+^, the band gap increases. According to this classification, as the B^I^ group is changed from Na^+^, to K^+^ to Rb^+^, the B^I^-*s* orbital energy increases, lifting the CBM. At the same time *p*-*s* coupling between B^III^ and X^VII^ is enhanced because of the expanded size of the B^I^X^VII^_6_ octahedra and reduced size of the B^III^X^VII^_6_ octahedra, elevating the VBM. In contrast, as the B^I^ group is changed from Cu^+^, to Ag^+^ to Au^+^, the band gap variation is dominated by a change of the VBM states. Interestingly, Ag-containing compounds show low VBM states and the largest band gaps because of the lowest *d* orbital among these three group IB elements. As In^+^ and Bi^3+^ ions have the same fully occupied outmost *s* shells as Pb^2+^, the band edge structure of Cs_2_InBiCl_6_ is similar to those of A^I^PbX_3_ compounds, and such double perovskites also shows a direct band gap. However, Tl-containing double perovskites exhibit lowered VBMs and substantially increased band gaps than those of Tl-contained perovskites because of the lower energy of Tl-*6s* than In-*5s* and the larger atomic size of Tl than In. As a consequence, Cs_2_AgSbI_6_, Cs_2_AgBiI_6_, and Cs_2_AuBiBr_6_ have appropriate direct band gaps of ~1.68, ~1.77, and ~1.83 eV, respectively. In contrast, Cs_2_InSbCl_6_ and Cs_2_InBiCl_6_ have ideal band gaps of ~1.02 and ~0.91 eV, similar to that of silicon (~1.14 eV), providing some potential high-performance OAMs. Despite similar results by Savory and co-workers of replacing Ag by ns^2^ ions such as In^+^ and Tl^+^ to create a direct allowed transition and to avoid the electronic mismatch in angular momentum of the frontier atomic orbitals between Ag^I^ and Bi^III^, these authors also pointed out that the introduction of these elements leads a narrowing of the band gaps, particularly for In [48]. Additional studies on Cs_2_NaLnCl_6_ (Ln = Y, Nd, Sm, Eu, Tb, Er, Yb) and Cs_2_NaYCl_6_: Ln^3+^ (Ln = Sm, Er) as upconversion luminescence materials have indicated that lanthanides can also be regarded as potential non-toxic alternatives [49–54].

**Figure 2.**
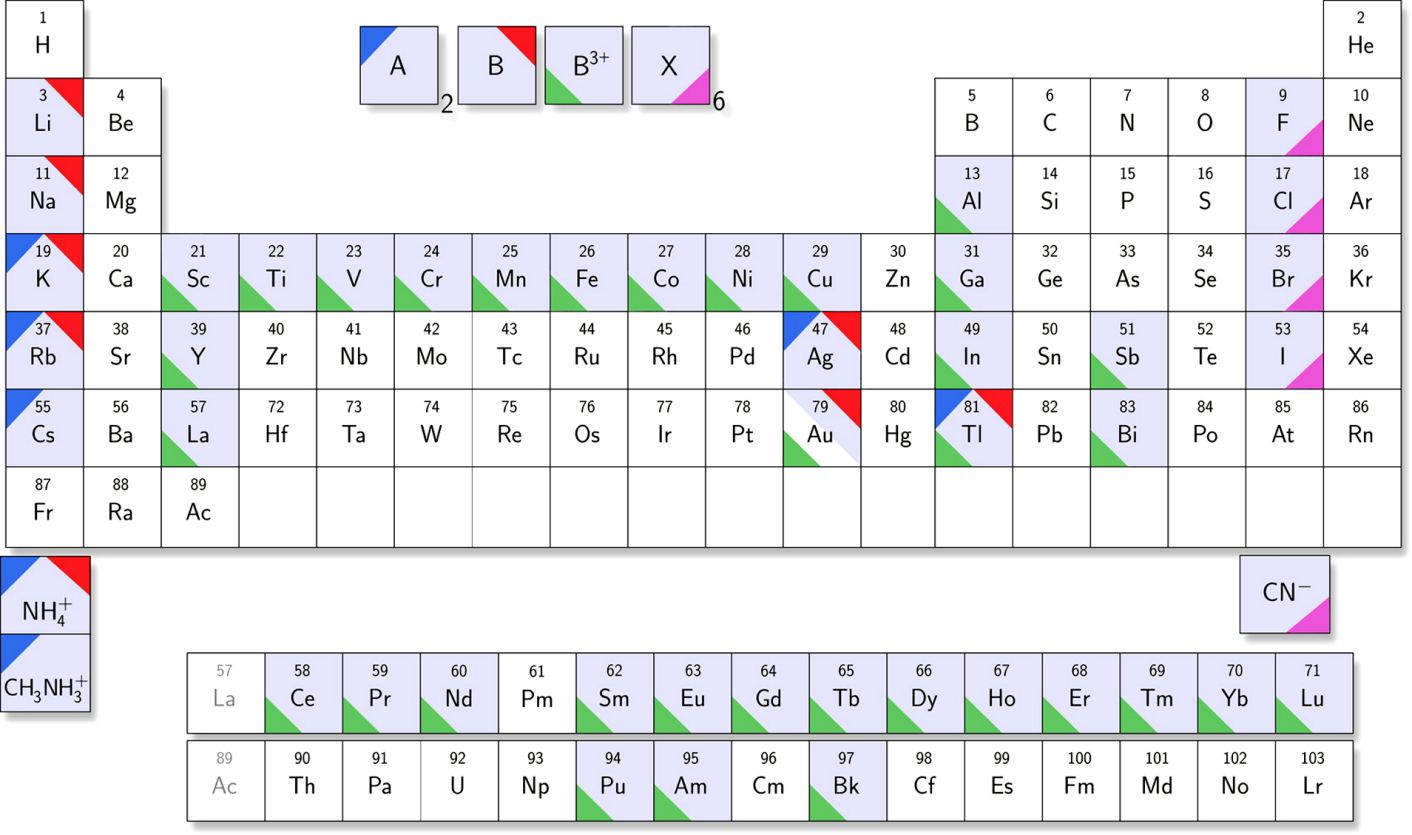
Elements forming halide double perovskites (elpasolites) with composition A_2_BB^3+^X_6_ [55].

Note: Copyright 2016, American Chemical Society.

Taking into consideration both homovalent lead substitution and heterovalent substitution, all the potentially less toxic alternatives, involving 7 A-site cations, 8 B^I^-site cations, 34 B^II^-site cations, and 5 X-site anions are summarized in Figure 2 [55]. Last but not least, although the alternatives in this section have been discussed with regard to double perovskites, these elements can also be used alone for atypical perovskites.

In recent years, tin-, germanium-, bismuth-, antimony-, copper-, and manganese-based PSCs have all been widely investigated in a number of laboratories. Among these PSCs, great progress has already been achieved for the tin-based PSCs. Table 1 presents a summary of the device performance of low-lead perovskite solar cells. In the following, we further focus on the detailed materials design, interfacial modification, fabrication, and device architecture of these low-lead PSCs. We also explain the component–structure–property relationships in these materials.

**Table 1.** Summary of device performance of low-lead perovskite solar cells.

Perovskite	Device Architecture	Short-circuit current (mA·cm−2)	Open-circuit voltage (V)	Fill Factor	Power Conversion Efficiency (%)	Year	Reference
CsSnI2.95F0.05+5%SnF2	FTO/TiO_2_/N719 Dye/Perovskite/ZnO	19.2	0.72	0.73	10.2	2012	60
CsSnI_3_ + 20 mol% SnF_2_	FTO/Compact TiO_2_/Mesoporous TiO_2_ /Perovskite/m-MTDATA /Au	22.7	0.24	0.37	2.02	2014	72
CsSnI_2.9_Br_0.1_	FTO/Compact TiO_2_/Mesoporous TiO_2_ /Perovskite /Spiro-OMeTAD+Li-TFSI+tBP/Au	24.2	0.22	0.33	1.76	2015	74
CsSnI_3_/ICBA	ITO/CuI/Perovskite/Fullerene/BCP/Al	12.3 ± 0.5	0.43 ± 0.06	0.39 ± 0.05	2.76	2015	62
CsSnI_3_/PCBM		8.9 ± 0.3	0.36 ± 0.03	0.54 ± 0.04	2.07		
CsSnI_3_/C_60_		11.6 ± 1.0	0.28 ± 0.03	0.43 ± 0.03	1.72		
CsSnI_3_	ITO/NiO_x_/Perovskite/PC_61_BM/Al	10.2	0.52	0.63	3.31	2016	77
CsSnI_3_ + 10 mol% SnCl_2_	ITO/Perovskite/PC_61_BM/BCP/Al	9.9 ± 0.6	0.50 ± 0.01	0.68 ± 0.01	3.4 ± 0.2	2016	68
Cs2SnI6 +Z907	FTO/TiO_2_/Dye/Perovskite/Pt	13.2	0.57	0.61	4.63	2014	78
Cs2SnI6 +N719		14.7	0.63	0.68	6.32		
Cs2SnI6 +multiple		16.9	0.62	0.66	6.94		
Cs2SnI6 +(with 3D PhC)		18.6	0.62	0.68	7.8		
Cs_2_SnI_6_	FTO/ZnO seed layer/ZnO nanorods/Perovskite/P3HT/Ag	3.2	0.52	0.515	0.86	2016	79
Cs2SnI6	FTO/TiO_2_/Perovskite/P3HT/Ag	5.4	0.51	0.35	0.96	2017	80
MASn_0.25_Pb_0.75_I_3_	FTO/Compact TiO_2_/Mesoporous TiO_2_ /Perovskite/Spiro-OMeTAD/Au	15.8	0.73	0.64	7.37	2014	83
MASnIBr_2_	FTO/Compact TiO_2_/Mesoporous TiO_2_ /Perovskite/Spiro-OMeTAD/Au	12.3 ± 0.5	0.82 ± 0 .03	0.57 ± 0.02	5.7 ± 0.2	2014	87
MASnI_3_	FTO/Compact TiO_2_/Mesoporous TiO_2_ /Perovskite /Spiro-OMeTAD+Li-TFSI+tBP/Au	16.8	0.88	0.42	6.4	2014	36
MASn_0.5_Pb_0.5_I_3_	FTO/Compact TiO_2_/Mesoporous TiO_2_ /Perovskite/P3HT/Ag/Au	20.0	0.42	0.5	4.2	2014	87
MAPb_0.85_Sn_0.15_I_3−y_Cl_y_	ITO/PEDOT:PSS/Perovskite/PC_61_BM/Ag	19.1 ± 0.2	0.76 ± 0.01	0.660 ± 0.008	9.8 ± 0.3	2014	88
MASnI_3_	ITO/ZnO/Perovskite/spiro-OMeTAD/Au	11.1	0.97	0.68	7.66	2015	56
MASnI_3_	FTO/Compact TiO_2_/Mesoporous TiO_2_ /Perovskite/spiro-OMeTAD/Au	21.4	0.32	0.46	3.15	2015	81
MASnI_3_	FTO/Compact TiO_2_/Mesoporous TiO_2_ /Perovskite/PTAA/Au	17.4	0.27	0.39	1.86	2016	90
MASnI_3_	FTO/Compact TiO_2_/Mesoporous TiO_2_ /Perovskite/P3HT/Au	4.3	0.50	0.49	1.12	2016	89
MASn_0.75_Pb_0.25_I_3_	ITO/PEDOT:PSS/Perovskite/PC_61_BM/C_60_/Ag	22.4 ± 0.5	0.82 ± 0.01	0.78 ± 0.01	14.4 ± 0.6	2016	98
FASnI_3_ + 20 mol% SnF_2_	FTO/Compact TiO_2_/Mesoporous TiO_2_ /Perovskite/Spiro-OMeTAD/Au	24.5	0.24	0.36	2.1	2015	95
FASnI_3_ + 10 mol% SnF_2_ + pyrazine	FTO/Compact TiO_2_/Mesoporous TiO_2_ /Perovskite/Spiro-OMeTAD/Au	23.7	0.32	0.63	4.8	2016	96
FASnI_3_ + 10 mol% SnF_2_ + diethyl ether	ITO/PEDOT:PSS/Perovskite/C_60_/BCP/Ag	22.1	0.47	0.61	6.22	2016	97
FASnI_2_Br	ITO/PEDOT:PSS/Perovskite/C_60_/Ca/Al	6.8	0.47	0.54	1.72	2016	99
	ITO/MoO_x_/Perovskite/C_60_/Ca/Al	2	0.44	0.55	0.47		
FASn_0.5_Pb_0.5_I_3_	ITO/PEDOT:PSS/Perovskite/C_60_/BCP/Au	21.9	0.70	0.66	10.2	2016	57
FA_0.75_Cs_0.25_Sn_0.5_Pb_0.5_I_3_		26.7	0.74	0.71	14.1		
MA_0.5_FA_0.5_Pb_0.75_Sn_0.25_I_3_	ITO/PEDOT:PSS/Perovskite/PC_61_BM/C_60_/Ag	22.8	0.79	0.78	14.06	2016	98
FASnI_3_	ITO/PEDOT:PSS/Perovskite/C_60_/BCP/Ag	17.8	0.33	0.68	3.98	2017	76
CsGeI_3_	FTO/Compact TiO_2_/Mesoporous TiO_2_ /Perovskite/Spiro-OMeTAD/Au	5.7	0.07	0.27	0.11	2015	37
MAGeI_3_	FTO/Compact TiO_2_/Mesoporous TiO_2_ /Perovskite/Spiro-OMeTAD/Au	4.0	0.15	0.3	0.2		
Cs_3_Bi_2_I_9_	FTO/Compact TiO_2_/Mesoporous TiO_2_ /Perovskite/Spiro-OMeTAD/Au	2.2	0.85	0.6	1.09	2015	106
MA_3_Bi_2_I_9_		0.5	0.68	0.33	0.12		
MA_3_Bi_2_I_9_Cl_x_		0.2	0.04	0.38	0.003		
MA_3_Bi_2_I_9_	FTO/TiO_2_/Anatase/Perovskite/HTL/Au	0.8	0.56	0.48	0.259	2016	111
	FTO/TiO_2_/Perovskite/HTL/Au	0.6	0.51	0.33	0.108		
	FTO/TiO_2_/Brookite/Perovskite/HTL/Au	0.6	0.53	0.3	0.094		
Cs_3_Sb_2_I_9_	FTO/Compact TiO_2_/Perovskite/PTAA/Au	0.3	-	-	<1	2015	116
MA_3_Sb_2_I_9_	ITO/PEDOT:PSS/Perovskite/PC_61_BM/ZnO Nanoparticle /Al	1	0.90	0.55	0.49	2016	118
Rb_3_Sb_2_I_9_	ITO/TIO_2_/Perovskite/Poly-TPD/Au	2.1	0.55	0.57	0.66	2016	117
MAPb_0.85_In_0.15_I_3_Cl_0.15_	ITO/PEDOT:PSS/Perovskite/PC_61_BM/Bphen/Ag	21.9	1.03	0.78	17.55	2016	119
MA_2_CuCl_x_Br_4−x_	ITO/PEDOT:PSS/Perovskite/PC_61_BM/Al	0.22	0.26	0.32	0.017	2016	129
MAPb_x_Mn_1–x_I_1+2x_Cl_2–2x_	ITO/PEDOT:PSS/Perovskite/PC_61_BM/Al	0.02	1.19	0.88	0.32	2016	130

Notes: ITO: Tin-doped indium oxide; PEDOT:PSS: Poly(3,4-ethylenedioxy-thiophene)-poly(styrene sulfonate); PC_61_BM: Phenyl-C_61_-butyric acid methyl ester; Bphen: Bathophenanthroline; m-MTDATA: 4, 4′, 4″-tris (N, N-phenyl-3-methylamino) triphenylamine; BCP: Bathocuproine; FTO: Fluorine-doped tin oxide; N719:(cisdiisothiocyanato-bis(2,29-bipyridyl-4,49-dicaboxylato) ruthenium(II)bis-(tetra -butylammonium); P3HT: Poly(3-hexylthiophene-2,5-diyl); Poly-TPD: Poly[N,N’-bis(4-butylphenyl)-N,N’-bisphenylbenzidine]; PTAA: Poly[bis(4-phenyl)(2,4,6-trimethylphenyl)amine]; Spiro-OMeTAD: 2,2’,7,7’-Tetrakis[N,N-di(4-Methoxyphenyl)aMino]-9,9’-spirobifluorene; tBP:Tri-butyl-phosphate; PhC: Photonic crystals.

## 3. Tin-based halide perovskites

Tin (Sn) possesses a similar outer electron shell structure to Pb, but shows a smaller ionic radius (~1.35 Å) than that of Pb (~1.49 Å), encouraging the replacement of Pb by Sn with no significant perturbation in lattice structure. Despite this similarity, Sn-based perovskites exhibit a subtle difference in chemical bonding and semiconducting properties compared to Pb-based perovskites due to a larger degree of orbital overlap in the Sn-I bond than the Pb-I one. Specifically, the outer ns^2^ electron configuration, with low ionization energy, is helpful for Sn-based perovskites to obtain large optical absorption, a low exciton binding energy (~18 meV), a high charge mobility (~10^2^–10^3^ cm^2^·V^−1^·s^−1^) and a narrow band gap (~1.3–1.4 eV). However, as a result of relativistic effects Pb-*6s* ^*2*^ shows a larger stabilization relative to that of Sn-*5s* ^*2*^. It is nevertheless relevant that, to date, Sn-based PSCs have not yet benefited from the same intensive research effort that has propelled Pb-based perovskites, due to their low PCEs and susceptibility of Sn to ambient atmosphere with regards to oxygen and moisture, which give rise to a structural transition, and a low defect formation energy, with the difficultly in forming pinhole-free films additionally limiting their viability. Very recently, CsSnI_3_, CH_3_NH_3_SnI_3_ (MASnI_3_), and (NH_2_)_2_CHSnI_3_ (FASnI_3_) have been widely utilized in PSCs and the best reported PCEs of devices employing Pb-free and Pb-reduced Sn-based perovskites now already approach ~7.66% [56] and ~14.1% [57], respectively. Following these three-dimensional (3D) Sn-based perovskites, we also discuss the emerging low-dimensional perovskites such as (HA)SnI_4_, (BZA)_2_SnI_4_ [58], and (PEA)_2_(FA)_n-1_Sn_n_I_3n+1_, which exhibit high stability, excellent film quality, and an abundant diversity in optoelectronic properties [59].

### 3.1. Cesium tin iodines

CsSnI_3_, a p-type semiconductor with a smaller direct band gap of ~1.3 eV (~1.55 eV for MAPbI_3_) and corresponding a higher theoretical *J*_SC_ of ~34.3 mA·cm^−2^ (~25.9 mA·cm^−2^ for MAPbI_3_), shows unique contra-indicated properties combining strong photoluminescence (PL) with high electrical conductivity. This perovskite also exhibits high carrier concentration (~10^17^ cm^−3^) and hole mobility (~585 cm^2^·V^−1^·s^−1^), as well as a large optical absorption coefficient and low exciton binding energy (~18 meV), confirming its suitability as a viable OAM. Nevertheless, the first 3D orthorhombic CsSnI_3_ reported was developed for use as a hole transporter material (HTM) in solid-state dye sensitized solar cells (DSSCs) with a high PCE of ~10.2% [60]. Initial studies have indicated that the use as either an OAM or an HTM can be controlled on the basis of carrier concentration and electrical conductivity. According to this relationship, CsSnI_3_ can be promoted as a HTM in the Sn-poor condition when a high concentration of acceptor defects such as Sn or Cs vacancies is present, thus easily enabling high p-type conductivity and forming deep-level defects that can further become electron-hole recombination centers with high energy. By contrast, CsSnI_3_ can be regarded as an OAM when in a Sn-rich condition [61]. As described by Marshall and co-workers, the excess SnI_2_ additive to as-synthesized CsSnI_3_ can result in a reduction of the background carrier density, which gives rise to recombination losses, significantly increasing the *J*_SC_. A related study on using derivatives of C_60_ with modified frontier orbital energies commercially available as the n-type ETM has shown it is possible to improve interactions at the heterojunction, giving rise to a large abrupt vacuum level shift across the interface and further promoting its PCE with an increased *V*_OC_ [62].

Furthermore, efficient strategies for adjusting PV performance have been developed in phase-selective preparation, interface band gap adjustment and fabrication processes. In some early studies it was observed that CsSnI_3_ exhibits four polymorphs; two polymorphs existing independently in the room temperature condition with the other two resulting from phase transition. Specifically, these polymorphs consist of a yellow one-dimensional double-chain phase (Y) [59], a black 3D orthorhombic phase (B-γ), a black cubic phase (B-α) derived from the Y phase transition when heated above 425 K, and a black tetragonal phase (B-β) at 426 K (instead of Y) [63–65]. Additional studies have shown that the different electronic properties of these polymorphs is strongly related to the structure of the inorganic cage and the formation of the perovskite octahedral network [66,67]. Among these polymorphs, B-γ-CsSnI_3_ is ideal for PV applications, mainly due to a high stability of more than a year under inert conditions and suitable band gap of ~1.3 eV (~2.55 eV for the Y phase). However, when exposed to air, moisture or organic solvents, this polymorph can easily transform into the Y phase, known as the best packing model and thermodynamically stable form for CsSnI_3_, with a total loss of its PV performance. Therefore, it is necessary to prevent B-γ-CsSnI_3_ from phase transition in order to improve both the PCE and stability.

Following progress in phase-selective preparation, incorporation of excess additives (SnF_2_, SnCl_2_ or C_60_) into CsSnI_3_ films can minimize the density of tin vacancy defects with high rates of defect-mediated charge carrier recombination. Such material shows a dramatically improved stability and greatly increased PCE without significant hysteresis and a reduced number of parallel degradation pathways [68]. Additional studies by Marshall and co-workers have demonstrated a PCEs reduction of ~30 and ~70% from the initial value after 7 h under continuous 1 sun illumination in the non-encapsulated condition and tested in ambient air at ~ 50 °C. This is almost the highest stability reported for non-encapsulated Sn-based PSCs and bodes well for the possibility of realizing Sn-based PSCs with a useful lifetime [68,69]. It has also been demonstrated that due to its greater covalency, SnCl_2_ is much more soluble in common solvents than SnF_2_, contributing to the ability to prepare films from solution at room temperature. The propensity for solid-state diffusion of tin chloride into fullerenes is likely to be larger compared to the iodide and bromide analogues due to the smaller size and lower mass of the chloride species. The generality of this doping mechanism is suitable for C_60_ and devices using a simplified architecture can reduce the fabrication costs from a commercial perspective as well [70–72].

As integrating different halogens into CsSnX_3_ (X = halogen), or reducing the size to the nanometer-scale can both significantly tune the optical band gap, an initial study on changing the halogen in CsSnX_3_ from I, to Br to Cl has obtained a blue-shift from ~1.3 to ~2.8 eV [73]. A further study, taking into consideration the phase-selectivity and halogen substitution as concurrent criteria, has demonstrated that the change from orthorhombic-phase CsSnI_3_ to cubic-phase CsSnBr_3_ not only results in an adjustment of the band gaps from ~1.27 to ~1.75 eV, but also manifests in a transition from metallic to semiconductor characteristic [74]. In addition to combining compositional adjustments with spatial confinement effects, CsSnX_3_ (X = Cl, Cl_0.5_Br_0.5_, Br, Br_0.5_I_0.5_, I) studies on perovskite nanocrystals have indicated that the luminescence occurs on pico- to nanosecond time scales via two spectrally distinct radiative decay processes: luminescence can be assigned to band-to-band emission, while radiative recombination is promoted by the confinement of electrons and holes at shallow intrinsic defects [75].

Differently from conventional solution fabrication technologies, a solid-state dewetting method was used to obtain a fine-grain structure, resulting in an increase of c(V_Sn_), reducing the carrier lifetime, but significantly increasing the *J*_SC_ and FF values [72]. Further study on a ‘multichannel interdiffusion’ method has highlighted multiple interdiffusion pathways for complete reaction with SnI_2_ [76]. On the whole, annealing stacked layers of aqueous solution deposited formamidinium iodide (FAI)/PEDOT:PSS and followed with an evaporated SnI_2_ layer avoids the solubility issue of SnI_2_ or Sn halides in most solvents and results in uniform FASnI_3_ films. More importantly, this method also allows the preparation of a flexible PSC assembled on polyethylene naphthalate (PEN)-ITO substrates with a PCE of ~3.12%. Despite such noteworthy PV performance and the progress in CsSnI_3_, further commercialization is restricted as a result of its thermodynamic instability. In contrast, Cs_2_SnI_6_, a defect variant of CsSnI_3_ where half of the octahedral Sn atoms are missing, creating discrete molecular [SnI_6_]^2−^ octahedral exhibits high-symmetry cubic structure with good air and moisture stability. This defect variant can also be regarded as a ‘vacancy ordered’ double perovskite with structure of Cs_2_Sn^4+^□I_6_ (where □ stands for vacancy). Surprisingly, Cs_2_SnI_6_ shows a diversity in PV applications on the basis of doping concentration. For example undoped Cs_2_SnI_6_ shows n-type performance with carrier mobility of ~310 cm^2^·V^−1^·s^−1^ whereas doping with excess Sn^2+^ (SnI_2_) yields a nearly identical carrier concentration of ~1 × 10^14^ cm^−3^ but associated with p-type semiconductor characteristics, with a low carrier mobility of ~42 cm^2^·V^−1^·s^−1^. Accordingly, initial studies have shown that Cs_2_SnI_6_ can also be used as a HTM in DSSCs, with the highest PCE reported already approaching ~7.80% for a device using a mixture of dyes [77,78]. Recent studies by Qiu and co-workers have also demonstrated that the advantage of being able to fabricate Cs_2_SnI_6_ in ambient air represents an important step toward the realization of low-cost, lead-free, and environmentally benign next-generation solid-state solar cells with high efficiency [79,80].

### 3.2. Methylammonium tin iodines

**Figure 3.**
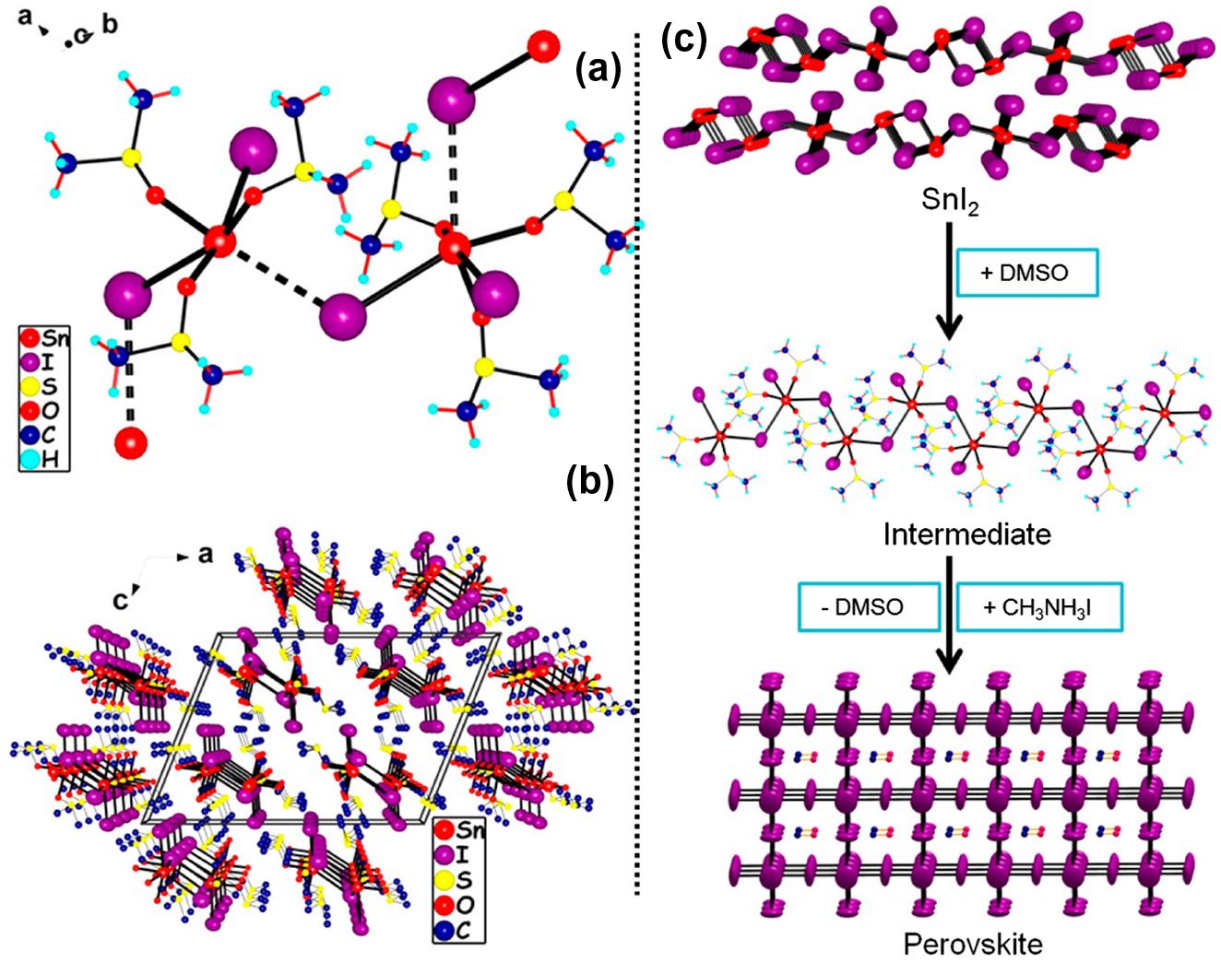
Crystal structure of SnI_2_·3DMSO, an intermediate compound in the MASnI_3_ film fabrication process. (a) The dimeric structure of the SnI(DMSO)_3_^+^ ions linked through the lone I^−^ ions. (b) The unit cell of SnI_2_·3DMSO in parallel view. (c) A schematic of the film formation of the MASnI_3_ perovskite film starting from SnI_2_ through the SnI_2_·3DMSO intermediate [80].

Note: Copyright 2015, American Chemical Society.

As organic analogues of CsSnI_3_, MASnI_3_, adopting orthorhombic phase, shows more diversity and adaptability in terms of structure and PV performance. Typically, such perovskites combine an ideal optical band gap (~1.3 eV), and high electron mobility (~2000 cm^2^·V^−1^·s^−1^) with low carrier concentration (~1 × 10^14^ cm^−3^). Based on these advantageous properties, MASnI_3_ has further been confirmed to provide n-type performance with a bulk electrical conductivity of ~5 × 10^−2^ S·cm^−1^ at room temperature and a corresponding Seebeck coefficient of ~260 mV·K^−1^, showing a great potential compared to MAPbI_3_ and other conventional semiconductors [36]. A uniform, high-coverage MASnI_3_ film has been achieved by Hao and co-workers through choosing dimethyl sulfoxide (DMSO) as the strongly coordinating/polar amide solvent and further controlling the crystallization process. In the method developed by these authors, a solvent-induced SnI_2_·3DMSO intermediate phase was used to promote homogeneous nucleation and to enable an adjustable perovskite film growth rate (Figure 3) [81]. The resulting films show an unprecedented *J*_SC_ reaching ~21.4 mA·cm^−2^ with a device carrier density one order of magnitude larger than that of the lead analogue. More importantly, this method represents important progress toward achieving improved perovskite morphology control in solution processed films.

Further studies on mixed Sn-Pb halide perovskites have also indicated that with increasing Sn content, these intermediate MASn_1-x_Pb_x_I_3_ perovskites show increasingly excellent optoelectronic performance compared to MAPbI_3_, such as perfectly balanced charge carrier transport, a narrow band gap (~1.17 eV), a high carrier concentration (~7.94 × 10^14^ cm^−3^), and a large carrier mobility (~2320 cm^2^·V^−1^·s^−1^), all in addition to a significant decrease in the usage of Pb [82–84]. Interestingly, this anomalous band gap narrowing is further theoretically identified to result from a competition between spin-orbit coupling (SOC) and the octahedral lattice distortion: the SOC causes a linear reduction with increasing x, while the lattice distortion causes a nonlinear increase, due to a composition-induced phase transition near *x* = 0.5 [85]. As a consequence, the best reported PCE of MASn_0.5_Pb_0.5_I_3_ is ~14.35%, highlighting their potential use as OAMs in energy conversion or detector devices.

Additional studies have further elucidated that the enhanced PV performance of MASn_0.5_Pb_0.5_I_3_ is also influenced by the concurrent effects of combining phase-selection and crystal symmetry. Generally, crystal symmetry can increase the band gaps in the sequence Pnma > I4cm > P4mm at x = 0.5. The binding energies of MASn_0.5_Pb_0.5_I_3_ decrease as the crystal symmetry decreases, implying a faster exciton dissociation with lower x and lower symmetry at an ambient temperature [86]. More importantly, these intermediate perovskites extend the light absorption into the near-infrared (∼1050 nm), making them ideal semiconductors for subcells to capture all photons and to further improve efficiency in perovskite/Si, perovskite/CIGS, perovskite/perovskite and perovskite/OPV tandem solar cells. Additionally, these perovskites can not only serve as the top subcell absorber for commercial solar cells, including Si and CIGS PVs, but also work efficiently as lower subcells, owing to highly tuneable band gaps extending down to the range of ~1.2–1.5 eV. However, some work is still needed to realize this approach on account of the restrictive incompatibility between device materials, for example the demands of depositing high-quality top cell layers without compromising the lower subcells.

The integration of different halogens into MASnX_3_ has also been explored allowing a tuning of the band gaps from ~1.30 to ~2.15 eV, along with a continuous contraction of the lattice parameters as the composition is changed from MASnI_3_ to MASnBr_3_ [87,88]. This strategy also allows control of the nucleation and growth process of these Sn-based perovskites as well as enhanced FF of devices at the same time. However, it should be noted that the active and interfacial layer resistances, the electrode resistance and the contact resistance may still reduce device performance. As a consequence, these approaches not only allow a reduced usage of Pb, mitigating ecological impact, but also enable higher PCE values than the pure Pb-based perovskites, enhancing their economic viability.

Similar to CsSnI_3_, high film quality with improved air-stability and reproducibility is a key factor to improve the PV performance of MASnI_3_. So far, co-evaporation, sequential evaporation [89], pulsed laser deposition (PLD) [56], low-temperature vapor-assisted solution process (LT-VASP) [90], and other advanced technologies have been developed to overcome the solubility issue of Sn halides and to control the preparation of selected crystal phases [91]. Taking into the consideration the advantages of both solvent engineering and conventional solution fabrication technology, Hao and co-workers have demonstrated that free carrier (electrons and holes) recombination is the dominant relaxation pathway in MASnI_3_ films. Accordingly a MASnI_3_ film with SnF_2_ doping effectively resulted in an increased fluorescence lifetime by up to ~10 times and gave diffusion lengths exceeding ~500 nm, making it possible to fabricate a planar structure device without a mesoporous electron-selective layer [92].

### 3.3. Formamidinium tin iodines

Although there is only a subtle difference in the A-site organic molecule from MASnI_3_, FASnI_3_ shows an attractive low band gap of ~1.41 eV, high temperature stability and good film quality because of the more rigid perovskite structure resulting from enhanced hydrogen bonding between larger sized HC(NH_2_)_2_^+^ cations and the Sn^2+^ matrix [93,94]. Indeed, FASnI_3_ exists only as one room temperature phase with a high phase transition temperature of ~473 K. In contrast, MAPbI_3_ shows a low temperature phase transition from tetragonal-phase to cubic-phase at 329 K, and FAPbI_3_ involves a black polymorph and a non-perovskite yellow polymorph associated with restricted PV performance. As previously mentioned, an initial study showed that the addition of excess SnF_2_ to an FASnI_3_ film could improve its PCE to a value of ~2.10% [95]. Further studies have indicated that integrating SnF_2_–pyrazine complex additives into FASnI_3_ films can result in the formation of a very smooth and dense film without any phase separation or plate-like aggregates, and lead to a reduction in charge recombination, thus yielding a high efficiency of ~4.8% with no hysteresis behavior in the J-V curves [96]. Recently a study has been carried out to combine a simple inverted device architecture with SnF_2_–diethyl ether additives to obtain dense, uniform, and full-coverage FASnI_3_ films, providing a narrow band gap of ~1.40 eV with the highest reported PCE of ~6.22% [97]. More importantly, due to the inverted device architecture and fullerene usage, these PSCs also demonstrate a small J-V hysteresis behavior and good long-term illumination stability under continuous 100 mW·cm^−2^ am 1.5G illumination, with only a ~5.86% drop over a period of ~2700 s, from an initial efficiency of ~5.80–~5.46%.

The tendency of the Sn^2+^ oxidation can be alleviated by incorporating FA cations to form mixed-cation perovskites with a narrow band gap of ~1.33 eV. The first reported PCE of MA_0.5_FA_0.5_Pb_0.75_Sn_0.25_I_3_ has already reached ~14.19%, and with 80% and 94% of the initial efficiency retained after 12 d storage in ambient (30–40% RH) conditions and 30 d of storage in an inert atmosphere, respectively. Further studies have shown that a highly efficient four-terminal all-perovskite tandem solar cell involving this perovskite yielded a great progress on its PCE with a value of ~19.08% [98]. A similar study on FASn_0.5_Pb_0.5_I_3_ PSC yielded a PCE of ~10.9%, while FA_0.75_Cs_0.25_Sn_0.5_Pb_0.5_I_3_ PSC exhibited an impressive PCE of ~14.1% and could also be well suited for a rear junction in a solution-processed tandem solar cell without the need for high temperature thermal processing, yielding a high PCE of ~20.5% [57]. For Pb- and Sn-based perovskites, the incorporation of FA and Br can also tune the band gaps as well as their stability in air [99].

### 3.4. Two-dimensional Sn-based perovskites

**Figure 4.**
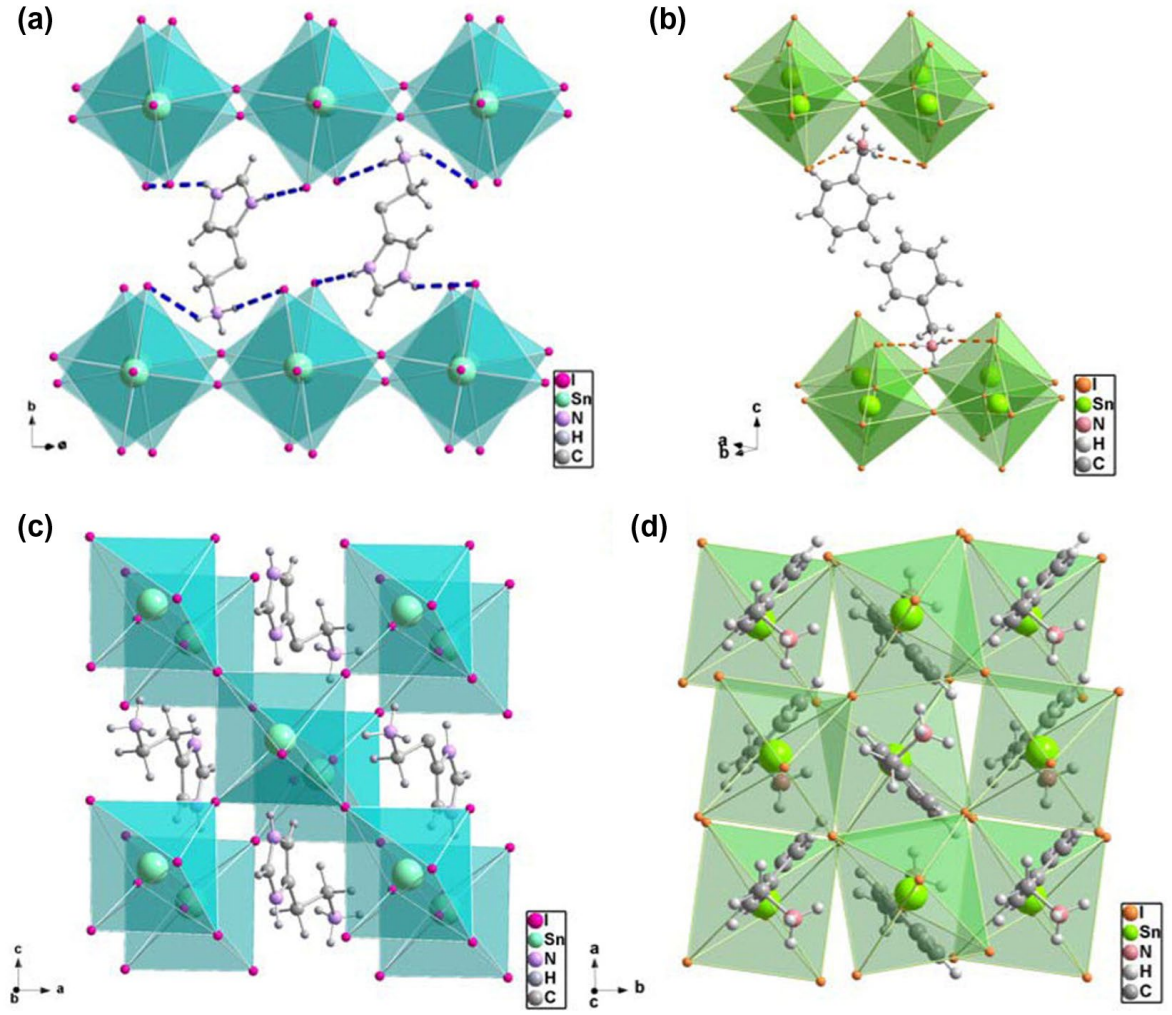
Coordination environment of (a) (HA)SnI_4_ and (b)(BZA)_2_SnI_4_; the Pb analogues of HA^2+^ and BZA^+^ have the exact same environments. (c) Top-down view of (HA)SnI_4_, ‘eclipsed’ conformation; (d) ‘staggered’ conformation of (BZA)_2_SnI_4_ [58].

Note: Copyright 2016, American Chemical Society.

Recently, two-dimensional (2D) perovskites have drawn more and more attention for PV applications, due to their superior structure and performance. Indeed, multilayered 2D (C_4_H_9_NH_3_)_2_(CH_3_NH_3_)_n-1_Pb_n_I_3n+1_ perovskites have been already developed with a high PCE of ~12.52% at *n* = 4, with good reproducibility and excellent operating stability [100]. Different from 3D perovskites, 2D perovskites have a layered crystal structure and offer greater synthetic functionalities, such as high stability and film quality, and tunability of optoelectronic properties by incorporating a wide array of organic cations into the 2D framework. 2D perovskites can be assembled by stabilizing single-layers separated by inorganic or organic cations, where each single-layer consists of corner-sharing octahedral [MI_6_]^4−^ units. These 2D perovskites main can be categorized into three types, according to the orientation of the cleavage plane of the ideal 3D cubic perovskite unit cell from which they are derived, namely (100)-oriented, (110)-oriented, and (111)-oriented systems. Typically, (100)-oriented 2D perovskites, with the formula of (RNH_3_)_2_(MA)_n−1_B_n_X_3n+1_, are formed by stacking n perovskite layers along the (100) direction of the parent 3D structure, separated by the long spacer ammonium cations, while in (110)-oriented perovskites, the octahedra are connected in a zigzag manner to form stacks of the perovskite along the (110) direction of the parent 3D structure. The (111)-oriented perovskites, normally using trivalent metal ions such as Sb^3+^ and Bi^3+^, have a formula of A_3_M_2_X_9_; these are discussed in more detail in sections 4 and 5. The 2D perovskites involving transition metal ions can also show magnetic properties, as discussed in section 8. An initial study on (HA)Pb_1-x_Sn_x_I_4_ (HA = histammonium) and (BZA)_2_Pb_1-x_Sn_x_I_4_ (BZA = benzylammonium) has demonstrated that these 2D perovskites can exhibit an anomalous band gap evolution with the structure transition, where the band gap decreases from ~2.05 to ~1.67 eV, as illustrated in Figure 4 [58]. Since hydrogen bonds between the N-H on the imidazolium group and the apical iodide are stronger than the other iodide atoms, (HA)SnI_4_ shows a stronger interaction than (BZA)_2_SnI_4_. Interestingly, due to the ‘wrong’ orientation of the structure (parallel to the substrate), BZA films showed poor PV performance, whereas HA films exhibited significantly better device performance because of the ‘correct’ layer orientation (perpendicular to the substrate). Additional studies on (C_6_H_5_CH_2_CH_2_NH_3_)_2_SnI_4_ ((PEA)_2_SnI_4_), (2-XC_6_H_4_C_2_H_4_NH_3_)_2_SnI_4_ (X = Br, Cl) [59], (PEA)_2_SnI_x_Br_4-x_ [101], and (PEA)_2_(FA)_n-1_Sn_n_I_3n+1_ [102] have shown suitable band gaps and relatively high charge carrier mobilities. Both (PEA)_2_SnI_4_) and (2-XC_6_H_4_C_2_H_4_NH_3_)_2_SnI_4_ further showed improved field-effect mobilities with values of ~0.030 and ~0.086 cm^2^·V^−1^·s^−1^, providing suitable material for organic–inorganic field-effect transistors (TFTs). Further studies on (PEA)_2_SnI_x_Br_4-x_ revealed a shorter decay lifetime as result of the greater spatial confinement of electrons and holes in the 2D structure, leading to a stronger Coulomb interaction than for the comparable 3D film, and to an increased probability of radiative recombination. In a recent (PEA)_2_(FA)_n-1_Sn_n_I_3n+1_ has been prepared with an orthorhombic structure with the *b*-axis perpendicular to the planes of the PEA bilayer, to form a continuous carrier transport pathway free of confinement barrier impediment [102]. As the encapsulating organic ligands and the compact perovskite film prevent oxygen ingress, such a 2D structure showed an enhanced thermodynamic stability suppressing the formation of Sn^4+^. As a result, the PSC showed a p-type behavior with a PCE of ~5.1 ± 0.6% and with ~96% of this initial value retained over ~100 h.

## 4. Germanium-based halide perovskites

Germanium (Ge^2+^) possesses a similar outer ns^2^ electron configuration (4s^2^) to Sn^2+^ (5s^2^) and Pb^2+^ (6s^2^), indicating that the Ge-based perovskites may exhibit analogous solid-state properties as Sn- and Pb-based ones. Its ionic radii is, however, much smaller than that of Sn^2+^ and Pb^2+^, leading to more diversity in structure and performance. As a result Ge-based perovskites display a higher ionic conductivity than others and can be classified into three major categories, namely 3D frameworks, where single Ge-I-Ge bridges form [GeI_6_]^4−^ corner-sharing octahedra that crystallize into the polar space group R3 m, 1D hexagonal systems, formed by [GeI_6_]^4−^ face-sharing octahedra where the [GeI_3_]^−^ units are connected by Ge-I_3_-Ge triple bridges and then stack one behind another down a (pseudo)hexagonal axis, and quasi-3D pseudohexagonal systems, which crystallize in the monoclinic space group P21, consisting of face-sharing [Ge_3_I_9_]^3−^ linear building blocks.

Initial studies have demonstrated that CsGeI_3_, MAGeI_3_, and FAGeI_3_ all show suitable band gaps with values of ~1.63, ~2.0 and ~2.35 eV and exhibit high stability, with CsGeI_3_ showing a higher stability of ~623 K compared to that of MAGeI_3_ and FAGeI_3_ (up to ~523 K). However, the highest PCEs of CsGeI_3_ and MAGeI_3_ reported remain low, at values of ~0.11% and ~0.20%, on account of factors including the formation of Ge^4+^ by oxidation during the synthesis procedures, poor solubility in polar solvents, strict control of the synthesis atmosphere, low quality films, a judicious choice of the HTL, and even the challenge of disproportionation [37,103]. An additional study on the optimized configurations of MAGeX_3_ has demonstrated that although Ge-based perovskites show crystallographic stability due to the tolerance factors, the weak absorption of MAGeI_3_ in the ultraviolet spectrum might be the main reason for its low PCE [104]. Another study has been carried out on the use of montmorillonite as bifunctional buffer layer to prevent charge recombination and to provide some corrosion protection, resulting in an improvement in the stability of MAGeI_3_ [105]. To obtain low-lead perovskites, a series of hybrid organic germanium perovskites with the formula of AGeI_3_ has been investigated and their Rashba splitting effect, stemming from their active lone pairs, has been demonstrated, together with a characterization of the associated structural distortions, direct and indirect energy gaps and strong nonlinear optical properties [102]. Several breakthroughs have been reported regarding the development of promising alternatives, including 3D CH_3_C(NH_2_)_2_GeI_3_ (MFOGeI_3_) with a direct band gap of ~2.5 eV, and 1D infinite chain structures such as, C(NH_2_)_3_GeI_3_ (GUAGeI_3_), (CH_3_)_3_NHGeI_3_ (TMAGeI_3_), and (CH_3_)_2_C(H)NH_3_GeI_3_ (IPAGeI_3_) with indirect band gaps of ~2.7 eV, ~2.5 eV, and ~2.8 eV, respectively. It has been noted that, since the largest size mismatch between Ge^2+^ and iodide leads to large polarizability and produces a strong SHG response, small cations such as Cs^+^, CH_3_NH_3_^+^, and HC(NH_2_)_2_^+^ can stabilize the distorted cubic perovskite structure, showing a strong dependence on the cation size.

## 5. Bismuth-based halide perovskite

Bismuth (Bi) is close to Pb in the periodic table and the Bi-*s* orbital shows quite similar features to the Pb-*s* orbital. This element also shows desirable semiconducting performance, as well as abundant structural diversity and low toxicity, marking it as a possible candidate to replace Pb. Bi-based perovskites crystallize with space group P3m1 and adopt a layered and vacancy-ordered structure with the formula unit of A_3_Bi_2_X_9_, where one third of the octahedral B^3+^ sites are vacant to maintain charge neutrality. Further integration of different organic groups into bismuth iodide octahedra has shown that these perovskites can form mononuclear or polynuclear inorganic frameworks of corner-, edge- and face-sharing bismuth halogen anions, leading to a wide range of structures and tunable optoelectronic properties. As an example, these perovskites can transform into zero-dimensional dimers of face-sharing BX_6_ octahedra (space group P63/mmc) when A-site cations are substituted with large organic molecules such as CH_3_NH_3_^+^. Despite these promising indications Bi-based PSCs show low PCEs due to their large binding energies, low-quality of crystallization, and weak interfaces at collector–perovskite heterojunctions, as well as high recombination rates, all of which restrict their potential for PV applications.

An initial study on the use of Cs_3_Bi_2_I_9_ and MA_3_Bi_2_I_9_ as OAMs was carried out in 2015, where it was demonstrated that MA_3_Bi_2_I_9_ showed a band gap of ~ 2.1 eV, with a corresponding efficiency of ~0.12% and enhanced air stability compared to MAPbI_3_ [106]. An additional study showed that the carrier density in MA_3_Bi_2_I_9_ can reach ~10^16^ cm^−3^, which is 7 orders of magnitude higher than in MAPbI_3_ (10^9^ cm^−3^). In the same study an improved PCE was achieved, reaching ~0.19% with little hysteresis, exhibiting a distinct advantage over Pb-, or Sn-based perovskites in solution-processable solar cells [110]. Further study has demonstrated that the optoelectronic properties of these perovskites are sensitive to the stoichiometry of the organic halide and the choice of salts, and that their film quality is also sensitive to experimental conditions and to desired/undesired colloids in the precursor solutions. Consequently, controlling the process of nucleation and growth, together with appropriate choice of substrate can result in improved crystal quality and final device performance [108]. Non-radiative recombination induced by defect states in the band gaps, and the effective masses of the charge carriers of Cs_3_Bi_2_I_9_ and MA_3_Bi_2_I_9_ by Pazoki and co-workers has been studied with a view to significantly improving both *V*_OC_ and *J*_SC_ [109]. Samples of MA_3_Bi_2_I_9_ PSC prepared on anatase TiO_2_ mesoporous layer showed good film coverage and a continuous smooth interface, reducing junction resistance as well as charge recombination, to values better than those of planar and brookite mesoporous PSCs [111]. However, to the best of our knowledge, only a few reports have explored the suitability of MA_3_Bi_2_I_9_ perovskites in planar or mesoporous device architectures, and no comprehensive study covering a variety of device architectures has been reported until now. Apart from these studies on MA_3_Bi_2_I_9_, an additional study on A_3_Bi_2_I_9_ (A = K^+^, Rb^+^, Cs^+^, NH_4_^+^, or MA^+^) has indicated that the band gaps are insensitive to the A-site cation size, owing to the hybridization of the Bi-6s/6p and I-5p states. This hybridization further involves the interplay of three factors: the chemical pressure due to the size of the A-site cations, the strength of H-bonds between H^+^ ions of the organic A-site cations and the I^−^ ions, and spin-orbit coupling (SOC) effects [107]. Among the materials studies, K_3_Bi_2_I_9_ showed promise for PV applications, with a band gap of ~1.98 eV.

Recently, Bi-based halide double perovskites have drawn more attention owing to their high stability. However, these double perovskites show wide band gaps of ~1.95–3.02 eV and the first double perovskite reported, Cs_2_NaBiCl_6_, is a colorless solid, which limits its use in PV applications. Fortunately, according to the radius–ratio rules and the calculated band gaps, the bottom of the conduction band is predominantly of antibonding Bi-6p/halogen-p character, and the top of the valence band is associated with Ag-*4d*/halogen-*p* antibonding orbitals. Thus, additions of Ag is appropriate to support the octahedral coordination of the halogens. Accordingly, it has been demonstrated that Cs_2_AgBiBr_6_ and Cs_2_AgBiCl_6_ with the space group Fm$\overset{¯}{3}$m showed band gaps of ~2.19 eV and ~2.77 eV, respectively, both being slightly smaller than those in the analogous lead halide perovskites [112]. Furthermore, although Cs_2_AgBiBr_6_ degraded over a period of weeks when exposed to both ambient air and light, these perovskites showed more stability when only exposed to air. Additional studies on Cs_2_AgBiBr_6_ showed an indirect band gap of ~1.95 eV and a long room-temperature PL lifetime of ~660 ns, which is higher than that in high-quality MAPbBr_3_, and suitable for use in tandem solar cells. It has been pointed out the lifetime of this perovskite is much shorter in powder form (~54 ns) than that in single crystal form (~145 ns) on account of the higher defect density and surface states originating from traps and/or surface-state emission [113]. Further study has shown that the band gap of Cs_2_BiAgCl_6_ is narrower than that of Cs_2_BiAgBr_6_ due to the smaller difference in energy between the Ag-d and Br-p levels as compared to the Ag-d and Cl-p levels, and the fact that the Br-p states are higher than the Cl-p states [114]. Despite these improved properties, the interaction between the Ag-*4d* orbitals and the *3p/4p* orbitals of the halide ion modified the valence band and led to an indirect band gap. Recently a study has been carried out to partially integrate Tl into Bi and Ag in compounds with the formula Cs_2_(Ag_1-a_Bi_1-b_)Tl_x_Br_6_ (0.003 < x = a + b < 0.075) with the aim of increasing the *6s* ^*2*^ and *6p* ^*0*^ orbital near the band edges of Cs_2_AgBiBr_6_ [115]. As a result, the band gaps arising from alloying with Tl are much lower than in the undoped material (~1.95 eV) or than in fully Tl-substituted compounds (~2.16 eV). Although dopants can form recombination trap states and thus decrease the carrier lifetime, these perovskites exhibited long-lived carriers, suggesting that the carriers can be efficiently extracted in PSCs.

## 6. Antimony-based halide perovskites

Antimony (Sb) shows less toxicity than Pb and exhibits a similar atomic structure and properties, as well as a lower exciton binding energy to Bi. Typically, Cs_3_Sb_2_I_9_ perovskites involve two structures: the 0D form (space group P_63_/mmc) and the 2D <111>-stacked layers form (P$\overset{¯}{3}$m1). Investigation of the layered form has shown a nearly direct band gap of ~2.05 eV, together with enhanced air-stability and a *V*OC of ~0.307 V with corresponding low PCE of below 1%, appearing to be the more promising form for PV applications. In contrast, the 0-D form showed a large indirect band gap of ~2.4 eV [116]. Further studies have shown that replacement of Cs by Rb can act significantly to template the formation of the desired layered phase, irrespective of processing methodologies, and also to improve charge transport properties [117]. Accordingly, Rb_3_Sb_2_I_9_ exhibited a large absorption coefficient of ~1 × 10^5^ cm^−1^ on the basis of p-to-p direct transitions and yielded the best device performance with a *V*_OC_ of ~0.55 V and a *J*_SC_ of ~2.12 mA·cm^−2^.

Similar to Cs_3_Sb_2_I_9_ and MA_3_Bi_2_I_9_, MA_3_Sb_2_I_9_ crystallizes in hexagonal-form with space group P63/mmc and shows a large absorption coefficient of ~10^5^ cm^−1^, together with a wide band gap of ~2.14 eV but with no exciton peak in its absorption spectrum. Furthermore, this perovskite can also be considered as 0D octahedral anionic metal halide units (Sb_2_I_9_)^3−^ surrounded by three MA^+^ cations and connected via a hydrogen bonding interactions, showing a strong preferential growth direction along the c-axis. A PSC using this material yielded a PCE of ~0.49% with low *J*_SC_ [118].

## 7. Indium-based halide perovskites

In is a post-transition metallic element located diagonally adjacent to Pb and commonly loses its three outermost electrons to form In^3+^ ions as a stable oxidation state. Owing to the good solubility of InCl_3_, partial substitution of Pb by In allowed synthesis of a superior MAPb_1-x_In_x_I_3_Cl_x_ with a substantially improved PCE of ~17.55% in a planar heterojunction [119]. Recently, Cs_2_InBiCl_6_ and Cs_2_InSbCl_6_ have been reported to crystallize with space group Fm$\overset{¯}{3}$m and to show suitable direct band gaps of ~1.02 and ~0.91 eV, as well as small effective masses of electrons and holes and thermodynamic stability [120,121]. Unfortunately, these perovskites are unstable against oxidation and other reduction–oxidation conditions, severely restricting their commercial potential. As a consequence, a recent study on A_2_B^I^B^III^X_6_ (A = K, Rb, Cs; B^I^ = Cu, Ag; B^III^ = Ga, In; X = Cl, Br, I) has been carried out to take advantage of the strong Cu(d)/Se(p)→Ga/In(s/p) valence-to-conduction band absorption spectra known from CIGS. The resulting samples show wide-range direct band gaps (from zero to ~2.5 eV), together with good thermodynamic stability [122]. At present the best alternatives reported are Rb_2_CuInCl_6_, Rb_2_AgInBr_6_, and Cs_2_AgInBr_6_ with direct band gaps of ~1.36, ~1.46, and ~1.50 eV, respectively.

## 8. Transition metal-based (Cu, Mn) halide perovskites

Transition metals such as Cu^II^ [123,124], Cr^II^ [125], Mn^II^ [126] and Fe^II^ [127,128] have been reported to extend significantly the field of synthesis of new perovskites for PV applications, and benefit additionally from physicochemical diversity and high crustal abundance. In contrast to the previously discussed perovskites, initial studies have indicated that because of their smaller ionic radii than Pb and other reported elements, these transition metal–perovskites have a layered structure that is isostructural to compounds of the Ruddlesden–Popper phase, such as K_2_NiF_4_.

Cu (electronic configuration 3d^9^ (t_2g_^6^ e_g_^3^)) shows a high stability in aerobic environments and possesses an ability to form compounds with a large absorption coefficient in the visible region. Additionally, 2D Cu-based perovskites also exhibit distinctive magnetic properties, whereby they behave like quasi-2D Heisenberg ferro-magnets. Studies on MA_2_CuCl_x_Br_4−x_ have demonstrated that ligand-to-metal charge transfer transitions can significantly tune the band gap, thus extending the optical absorption to the near-infrared, providing optimal spectral overlap with solar irradiance. Based on this approach samples of MA_2_CuCl_2_Br_2_ and MA_2_CuCl_0.5_Br_3.5_ have been prepared showing wide-range band gaps (~2.48 eV and ~1.80 eV, respectively) and with low PCEs of ~0.017% and ~0.0017%, respectively [129]. This study represents the first time that 2D ‘green’ perovskites based on transition metal have been used for PV applications.

Additionally a recent study has also demonstrated that MAPb_x_Mn_1–x_I_1+2x_Cl_2–2x_ (x = 0.1–1.0) showed suitable band gaps of ~1.54–1.56 eV, and a device based on this material using an inverted planar architecture was fabricated at low temperature, for which an outstanding PCE of ~0.32% was reported [130].

## 9. Conclusions and outlook

In this review we have briefly summarized the state of the art regarding low-lead perovskites in photovoltaics. In the past few years, by using either homovalent or heterovalent lead substitution, several promising low-lead alternatives have been proposed and theoretically modeled with the aim of reducing the lead-related toxicity in current benchmark lead halide perovskites. Examples of Sn-, Ge-, Bi-, Sb-, In-, and early transition metal-based PSCs have been synthesized and their photovoltaic responses have been investigated. Of these PSCs, however, only the Sn-based lead-free perovskite showed a promising PCEs when carefully prepared and tested. The PCEs of all these perovskites are still low, and far from the efficiency requirement for commercial application. Although partial substitution of Pb with non-toxic elements has yielded samples with improved PCE, such as ~14.35% for FASn_0.5_Pb_0.5_I_3_ and ~17.55% for MAPb_0.85_In_0.15_I_3_Cl_0.15_, these perovskites still contain Pb. It is sobering to note that at present the best PCE of a fully lead-free PS reported shows a PCE of only ~6.22% (for FASnI_3_ containing 10 mol% SnF_2_). Thus, fabrication of new lead-free perovskites and investigation of approaches to enhance PV performance still remain open challenges. Size effects on the optoelectronic properties of these lead-free perovskites needs to be investigated.

It is important to better understand the crystallization process for these lead-free perovskites, especially as in many cases the crystallization processes are quite different to those of lead halide perovskites. In this regard progress is highly desired toward achieving improved perovskite morphology control in solution-processed highly efficient lead-free PSCs. At the same time, improvement or development of fabrication technologies for large scale production is important as the first step for industrial application. Although the correlation of some physical properties, such as dielectric behavior, ferroelectricity, band gap, optical absorption, and electrical conductivity with PV performance has been studied, the structure–properties relationships are still unclear for the lead-free perovskites. It is imperative also to understand the excited-state dynamics in perovskites for further optimization of PSCs, as charge generation and recombination processes play important roles in dictating optoelectronic performance. Recent work using broadband transient absorption and time-resolved fluorescence spectroscopy has demonstrated comparable carrier lifetime and diffusion length for MASnI_3_ with MAPbI_3_.

Degradation of perovskite materials is also a key problem to be addressed. Hybrid lead halide perovskites are also known to degrade due to light, moisture heat and phase transition, leading to unstable operation of PV devices. An understanding of the degradation mechanisms will be helpful to enhance long-term stability, which will be a significant criterion for the commercialization of perovskite solar cells. Recently, some remarkable progress in the synthesis of stable perovskite has been made, including the use of a bilayer to avoid phase transition caused by ion migration or other inert conditions. Examples of newly synthesized stable perovskites include B-γ-CsSnI_3_ (stable for more than a year under inert conditions), Cs_2_SnI_6_ (air-stable), Cs_3_Bi_2_I_9_ (air-stable), FASnI_3_ (only a single stable phase over a broad temperature range up to 473 K), and Rb_3_Sb_2_I_9_ (thermally stable up to 523 K), as well as double perovskites such as Cs_2_InBiCl_6_, Cs_2_InSbCl_6_, and Cs_2_BiAgBr_6_ (X = Cl, Br, I). Additionally, MA_3_Bi_2_I_9_ has been reported as being stable for a long time (5 weeks) even after storage in ambient air. Moreover, Cs_2_BiAgCl_6_ does not show any noticeable changes after 3 weeks in ambient air, while degradation in Cs_2_BiAgBr_6_ is observed only upon light exposure for 2 weeks.

Despite these austere challenges, including unclear component–structure–property relationships, low-efficiency, poor stability upon prolonged exposure to light, humidity, and high temperatures, and compatibility with large-scale manufacturing routes, some impressive progress in low-lead PSCs has already been made. We are confident that lead must, and can, be completely replaced by nontoxic elements in PSCs, and that this can ultimately be done without compromising their efficiency and stability.

## Disclosure statement

No potential conflict of interest was reported by the authors.

## Funding

This work was financially supported by the International Projects of Cooperation and exchanges NSFC [grant number 51561145007], the Natural Science Foundations of China [grant number 51702038], the Israel Science Foundation ISF-NSFC program, the National Energy Novel Materials Center [grant number NENMC-II-1705], and by the Ministry of Science & Technology, P. R. China: Sino-Italy International Cooperation on Innovation [grant number 2016YFE0104000].
